# Role of visual and non-visual opsins in blue light–induced neurodegeneration in *Drosophila melanogaster*

**DOI:** 10.3389/fpubh.2025.1644780

**Published:** 2025-12-01

**Authors:** Marina Piacenti-Silva, Samuel de Mattos Alves, Hulder Henrique Zaparoli, Marcela de Oliveira, Juliano Morimoto, Carolina L. Zilli Vieira

**Affiliations:** 1 Department of Physics and Meteorology, School of Sciences, São Paulo State University–UNESP, Bauru, São Paulo, Brazil; 2 Institute of Biosciences of Botucatu, São Paulo State University–UNESP, Botucatu, São Paulo, Brazil; 3 Institute of Mathematics, University of Aberdeen, King’s College, Aberdeen, United Kingdom; 4 Graduate Program in Ecology and Conservation (PPGECO), Universidade Federal do Paraná, Curitiba, Brazil; 5 Department of Environmental Health, Harvard T.H. Chan School of Public Health, Boston, MA, United States

**Keywords:** blue light, environmental stress, rhodopsins, neurodegeneration, mental health, model systems

## Abstract

**Introduction:**

Light plays a key role in regulating circadian rhythms and downstream physiological and behavioural functions. However, excessive exposure to artificial blue light (450–500 nm) can disrupt sleep, metabolism and neural integrity. Visual opsins mediate light-dependent signalling, but organisms also express non-visual opsins whose roles in blue-light-induced neural stress are not well understood.

**Methods:**

We used Drosophila melanogaster knockout lines lacking either visual rhodopsin 1 (Rh1^1^) or non-visual rhodopsin 7 (Rh7^1^), alongside wild-type (w^1118^) controls. Flies were continuously exposed to 488 nm blue light (1,320 lux; 1,120 μW·cm^−2^) from egg deposition until they were 20 days old. DNA damage (γ-H2Av immunostaining) and vacuole formation were quantified in brain regions associated with sensory processing and neurotransmission.

**Results:**

Rh1^1^ flies exhibited the highest levels of DNA damage and vacuolisation compared to the w^1118^ and Rh7^1^ lines. These effects were most pronounced in neuropils linked to sensory integration and synaptic activity.

**Discussion:**

Our findings demonstrate that the visual opsin Rh1 plays a predominant role in blue-light-induced DNA damage and neurodegeneration in the Drosophila central nervous system. This suggests that it is visual, rather than non-visual, opsins that mediate the neurotoxic effects of exposure to artificial light.

## 1 Introduction

Light is an environmental stimulus of primary importance to all forms of life, playing a fundamental role in the regulation of circadian rhythms and influencing a wide range of physiological and behavioral functions in living organisms (1, 2). However, with the increasing use of electronic devices, human exposure to light has increased significantly. For many people, this means that overall exposure to light from electronic devices can now total 8–10 h a day versus 3–5 h in earlier decades (3). The evening period is particularly critical because many people now extend their device use well into the hours before sleep, which significantly increases exposure to blue-enriched light (4–9).

Blue light is a high energy electromagnetic radiation in the wavelength range of 380–500 nm, known to be able to penetrate ocular tissues and reach the retina and deeper brain regions (10). Modern electronic devices such as smartphones, tablets, and laptops emit blue light with spectral peaks between 445 and 455 nm. Peak irradiance at 450 nm reaches up to 0.00526 mW/cm^2^ on laptops and 0.00102 mW/cm^2^ on smartphones under typical usage conditions—levels that, while below sunlight intensity, raise concerns about cumulative exposure and its long-term effects on ocular and neural health (11). In humans, prolonged exposure to blue light has been associated with retinal phototoxicity, circadian rhythm disruption, and neurodegeneration (12–14). Evidence from experimental and clinical studies suggests that even moderate levels of artificial light exposure (100–1,000 lux), especially in the evening, can suppress melatonin production, disrupt sleep and other biological processes controlled by the body’s circadian clock, and cause adverse effects, including mental disorders such as depression, anxiety, bipolar disorder and self-mutilation (15), and metabolic disorders such as cardiovascular disease and cancer (4–9, 16).

Although direct evidence in humans remains limited, these findings in animal models underscore the need to better understand the biological consequences of chronic blue light exposure. Prolonged exposure to blue light can induce early puberty in male rats, suppress spermatogenesis, and impair testicular integrity (17), highlighting the broader physiological effects of light exposure. Studies in both cell culture and animal models have also shown that blue light can accelerate aging, significantly reduce lifespan, and promote molecular stress responses in neural tissues, including oxidative stress and DNA damage (9, 14, 18).

The main photoreceptor shared between invertebrates and mammals are opsins, photosensitive membrane proteins associated with a chromophore, that change conformation from a resting state to a signaling state during the phototransduction process after the absorption of a photon (19, 20). In humans, rod photoreceptors contain rhodopsin (OPN2), which is responsible for twilight vision, while cone photoreceptors contain opsins (OPN1), which are responsible for daytime vision and can be divided into four subgroups according to their absorption spectra: long (LW or red), short 1 (SW1 or UV/violet), short 2 (SW2 or blue) and medium (MW) (20, 21). In addition to the visual opsins, animals also express non-visual opsins such as melanopsins (Opn4); encephalopsins or panopsins (Opn3); neuropsins (Opn5) and the retinal photoisomerase G protein-coupled receptor (RGR) (20, 22–24). These non-visual opsins are present in several structures besides the retina, including the brain, testicles, liver, skin, spinal cord and lungs, suggesting an extra-retinal photomodulation (22). *Drosophila* has seven opsin genes (Rh1, Rh2, Rh3, Rh4, Rh5, Rh6 and Rh7) (20, 25), which encode their corresponding proteins (rhodopsins). Rh1’s predominant expression in the outer photoreceptors occurs directly when exposed to environmental light (26). In contrast, Rh7 is expressed in the central brain, particularly in circadian pacemaker neurons, and has been proposed to mediate non-visual responses to light (27). However, its role in light-induced neurodegeneration remains poorly understood.

Studies in animals with ablated eyes or retinal degeneration show that physiological responses to light—such as pupillary reflexes, adjustments in circadian rhythms, early mortality and brain neurodegeneration (remain when the light source is removed), raising questions about the mechanisms of light perception in the organism (9, 18, 22, 28–30).

Advancements in the field have revealed the involvement of non-visual opsins in a variety of physiological functions, including the regulation of circadian rhythms (31), hormonal secretion (32), thermoregulation (33), and neuronal activity (34). For instance, Opn4 plays a central role in circadian entrainment through light detection in retinal ganglion cells; however, its expression in other tissues suggests the presence of additional functions (35). A similar observation has been made with Opn3 and Opn5, which have been implicated in metabolic regulation and neuroendocrine signaling (33, 36). The precise phototransduction mechanisms of these opsins in extra-retinal tissues remain to be elucidated; however, experimental models have demonstrated that these proteins can respond to light stimuli, even in the absence of ocular input, thereby supporting the hypothesis of peripheral light sensing (37). However, the mechanisms through which these non-visual opsins, particularly in invertebrate models, contribute to the physiological effects of light exposure, especially in the context of neurodegeneration, remain to be elucidated.

Animal models such as the *Drosophila melanogaster* fly have been used to investigate the role of different opsins due to functional homology with human photoreceptive cells (9, 38). of which we highlight two main ones: rhodopsin 1 (Rh1), encoded by the ninaE gene, present in the outer photoreceptor cells of the eye and orthologous to human OPN4 (39), and rhodopsin 7 (Rh7), present in the central brain in a subset of circadian pacemaker neurons (25, 39). Previous studies have shown that excessive activation of Rh1 by high-intensity or prolonged light exposure can lead to retinal degeneration in *Drosophila*, characterized by photoreceptor cell death and structural damage (40).

In this investigation, Rh1 and Rh7 were selected as the subjects of study due to their status as the most well-characterized visual and non-visual opsins, respectively, in the *Drosophila* model. Rh1 is the most abundantly expressed rhodopsin in the retina and is essential for phototransduction in the outer photoreceptor cells. Conversely, Rh7 is uniquely expressed in the brain, particularly in neurons involved in circadian regulation, and is considered the only known non-visual opsin in *Drosophila*. This makes Rh7 a compelling candidate for evaluating extra-retinal light effects on the nervous system.

In this study, the primary objective was to evaluate the role of visual and non-visual opsins in the central nervous system of *Drosophila melanogaster* following exposure to blue light. To this end, we employed genetically modified flies lacking Rh1 and Rh7, and we conducted a comprehensive analysis of DNA damage and vacuole formation. These phenomena are well-established indicators of neurodegeneration, and our study sought to elucidate the underlying mechanisms through which these light-sensitive proteins influence the brain’s response to electromagnetic radiation. The study yielded direct evidence for the differential contribution of visual (Rh1) and non-visual (Rh7) opsins to the negative effects of blue light exposure on the nervous system, underscoring their potential as targets in neuroprotection strategies.

## 2 Methods

**Figure 1:**
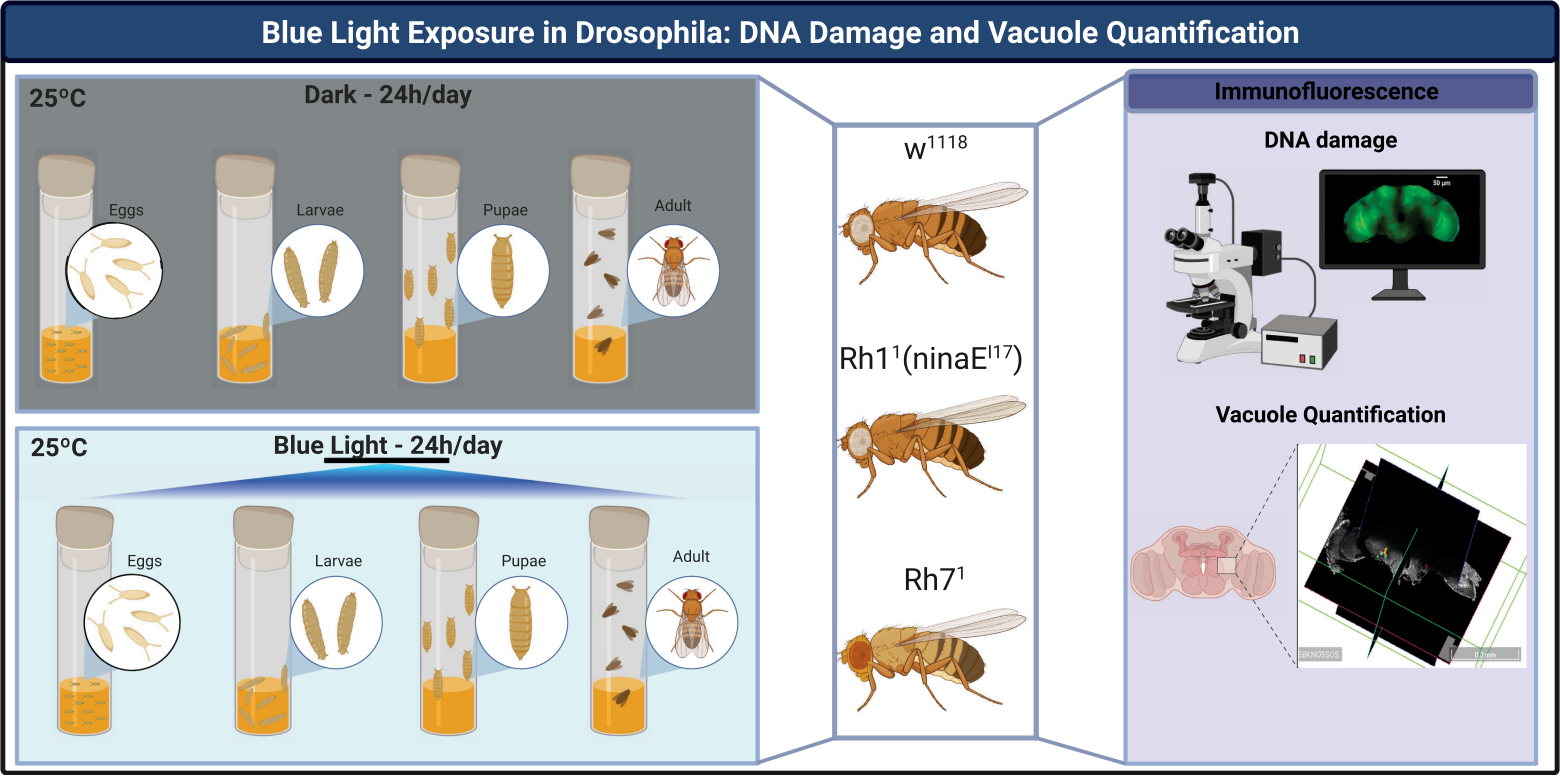
Experimental workflow of blue-light exposure and analysis in *Drosophila melanogaster*. Flies [w^1118^, Rh1^1^ (ninaE^I17^), and Rh7^1^] were reared at 25 °C and 70% relative humidity under either constant darkness or continuous blue light exposure (488 nm, 1,320 lux, 1,120 μW cm^−2^). Development from egg to adult occurred entirely under the assigned condition. Adult flies were analyzed at 20 days post-eclosion. Experimental outcomes included: (i) immunofluorescence staining to detect DNA damage (γ-H2Av, red), nuclei (Hoechst, blue), and actin fibers (Phalloidin, green); and (ii) volumetric quantification of brain vacuoles using 3D reconstruction software. Created with BioRender https://biorender.com/b4wjkjn.

The experimental workflow in Figure 1 illustrates the key steps of this study, from fly stock maintenance and experimental group assignment to outcome measurements in *Drosophila melanogaster*, including DNA damage and vacuole quantification analyses (Figure 1).

### 2.1 Fly stocks and experimental groups

The strains used in this study were: Rh1^1^(ninaE^I17^)—BDSC#5701; FlyBase ID:FBal0013022; Rh7^1^—BDSC#BL76022; FlyBase ID: FBal0323541; and w^1118^. Strains Rh1^1^ (ninaE^I17^) and Rh7^1^, represent flies in which the respective genes (ninaE and Rh7) were ablated with a loss-of-function allele (39). Stock flies were maintained in bottles and vials containing cornmeal agar in an incubator (Tritech Research Inc.—standard DigiTherm) at 25 °C and 70% relative humidity under a 12:12 h light–dark cycle with ambient broad-spectrum white light (400–700 nm; 1,216 lux). The lights were turned on at 9:00 a.m. and turned off at 9:00 p.m. Approximately 10 pairs of adult flies were placed per vial (cornmeal agar) for oviposition and kept in an environmental chamber. After 72 h, parental flies were removed to avoid overlapping generations. From egg deposition (Day 0) onward, vials were assigned to blue-light or dark groups and maintained at 25 °C and 70% relative humidity. The blue-light group was exposed continuously (24 h/day) at 488 nm (1,320 lux; 1.120 μW·cm^−2^); thus, development from egg to adult occurred entirely under the assigned condition. Upon eclosion, adults were allowed to mate for 48 h and were then separated by sex. Blue-light exposure continued uninterrupted for 20 days. This timeframe is consistent with previous *Drosophila* studies of chronic blue-light exposure that observed neurodegenerative changes, lifespan reduction and increased neuronal damage over similarly extended periods (18, 41, 42). Blue light exposure was maintained without interruption from day 0 to day 20, resulting in a cumulative dose of approximately 1.94 × 10^3^ J/cm^2^ at the sample plane.

Control vials were kept in constant darkness (24 h D/D) under otherwise identical conditions.

We use constant darkness as the control condition to exclude the confounding effects of light exposure and isolate the specific effects of blue light.

### 2.2 Irradiation system

The blue light irradiation system consisted of eight light-emitting diodes (LED Luxeon Rebels), which were powered by an external source. Intensity, lux, and spectral profile measurements were obtained using a power meter (Thorlabs PM100D), a light meter (Extech LT300), and a spectrometer (Thorlabs CCS200), respectively. The exposure parameters for the blue light (*λ* = 488 nm) were 1,320 lux and a power of 1,120 μW/cm^2^. These parameters were chosen based on previous studies (18, 41–45).

### 2.3 Immunofluorescence

To assess neuromorphological changes, we examined the *Drosophila* central brain (46–49). After 20 days of light exposure, flies were anaesthetized and fixed in 4% formaldehyde for 3 h at room temperature and washed three times for 20 min with PBS. Brains were dissected and incubated overnight at 4 °C under rotation in PBS/0.5% Triton X-100 + 2% BSA. Subsequently, primary antibodies were incubated at 4 °C with rotation. After ~24 h, brains were washed three times for 20 min with PBS/0.5% Triton X-100 + 2% BSA and incubated with secondary antibodies for 2 h at room temperature with rotation. Tissues were then washed three times for 20 min with PBS and mounted on slides using Vectashield (50). The primary antibody used to detect DNA damage was mouse anti-ɣH2AV (1:40 DSHB). This antibody binds to the phosphorylated variant of histone H2A, i.e., DNA double-strand breaks. The secondary antibody anti-mouse Alexa 647 (1:1000) was used to detect the primary antibody. Hoechst (497 nm—blue) was used for staining nuclei and Phalloidin (546 nm—green) for actin fibers. Images were obtained in 3 μm sections using a Leica SP8 confocal microscope with a 63× oil immersion objective. Approximately 100 sections were taken for each brain, and laser, filter, and gain settings were kept constant for all experiments. On average, 3 biological replicates (*n* = 3 animals per genotype × condition) were analyzed.

### 2.4 DNA damage quantification

Quantification of DNA damage in the samples was performed by counting γ-H2Av positive cells in the images. This was performed using Fiji Image J and 3D Slicer software. First, the images for γ-H2Av positive cells were merged with the images of cell nuclei using the merge channels tool in Fiji. Then, in this new image, the γ-H2Av positive cells that overlapped with the nuclear labelling were detected. Segmentation of the coincident cells was performed using the watershed segmentation method. This method is widely used in the analysis of cellular images to segment specific regions, such as nuclei, cells or organelles in an image. In general, the method is based on topography concepts and uses pixel intensity as a three-dimensional representation to partition the image into distinct regions corresponding to cellular objects and thus perform their segmentation (51, 52). Finally, the number of segmented cells was counted and cell damage quantified.

### 2.5 Vacuole quantification

We quantified vacuoles using an adapted version of the protocol for analyzing 3D neurodegenerative vacuoles in *Drosophila* (53). Images Z-stacks were first converted into RGB colour using Fiji Image J. The three-dimensional geometric segmentation and analysis was performed using the free software Webknossos. Segmentation was performed for selected layers of the Z-stacks and volume interpolation was performed to record the vacuole regions within the segmentation layers. Unstained areas were identified as vacuoles. Vacuoles located on the retina and any tissue damage resulting from sample processing were not considered. Quantification was performed blinded to genotype and condition. Vacuoles in the central brain were registered, and their qualitative and quantitative information was exported as 3D meshes in CSV files. The total number of sample vacuoles and the percentage of total brain volume occupied by vacuoles were determined using the Python 3.9 (Numpy-STL package).

### 2.6 Statistical analysis

Statistical analyses were performed using GraphPad Prism 8. Data are presented as mean ± SD. Group comparisons were carried out using a two-way ANOVA followed by a Tukey’s multiple comparisons test for γ-H2Av quantification and a Mann–Whitney or Kruskal–Wallis test followed by a Dunn’s *post hoc* test for vacuole quantification. Statistical significance was set at *p* < 0.05.

## 3 Results

### 3.1 Blue light exposure leads to increased DNA damage

**Figure 2:**
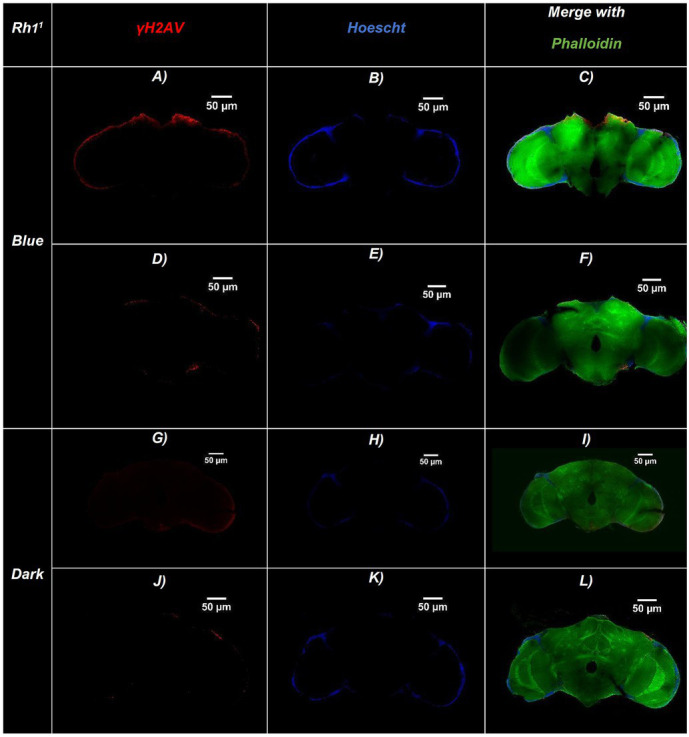
Representative confocal images of adult *Drosophila* Rh1^1^ (ninaE^17^) brains exposed to blue light or maintained in darkness. Brains were immunostained for DNA damage (γ-H2Av, red), nuclei (Hoechst, blue), and actin filaments (Phalloidin, green). **(A–F)** Brains exposed to continuous blue light (24 h/day for 20 days)—**(A,D)** γ-H2Av channel, **(B,E)** Hoechst channel, **(C,F)** Merged. **(G–L)** Brains maintained in constant darkness (24 h D/D): **(G,J)** γ-H2Av channel, **(H,K)** Hoechst channel, **(I,L)** Merged. Merged panels **(C,F,I,L)** show colocalization of all three markers, allowing visualization of brain structure and DNA damage. Brains exposed to blue light display a higher number of γ-H2Av–positive nuclei (red), indicating increased DNA damage compared to dark controls. Scale bars: 50 μm (all panels).

**Figure 3:**
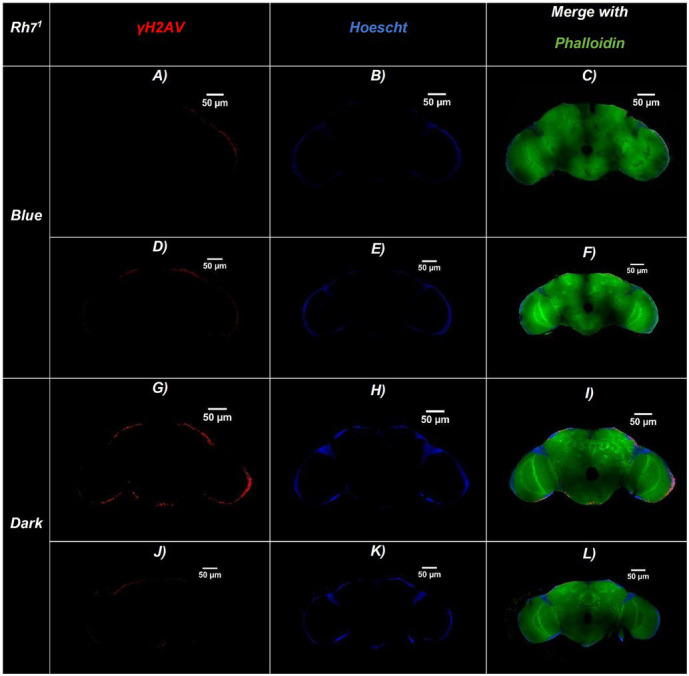
Representative confocal sections of adult *Drosophila* Rh7^1^ brains under blue-light or dark conditions. Brains were immunostained for DNA damage (γ-H2Av, red), nuclei (Hoechst, blue), and actin filaments (Phalloidin, green). **(A–F)** Brains exposed to continuous blue light (24 h/day for 20 days)—**(A,D)** γ-H2Av channel, **(B,E)** Hoechst channel, **(C,F)** Merged. **(G–L)** Brains maintained in constant darkness (24 h D/D): **(G,J)** γ-H2Av channel, **(H,K)** Hoechst channel, **(I,L)** Merged. Merged panels **(C,F,I,L)** show colocalization of all three markers, allowing visualization of brain structure and DNA damage. Brains exposed to blue light display a higher number of γ-H2Av–positive nuclei (red), indicating increased DNA damage compared to dark controls. Scale bars: 50 μm (all panels).

**Figure 4:**
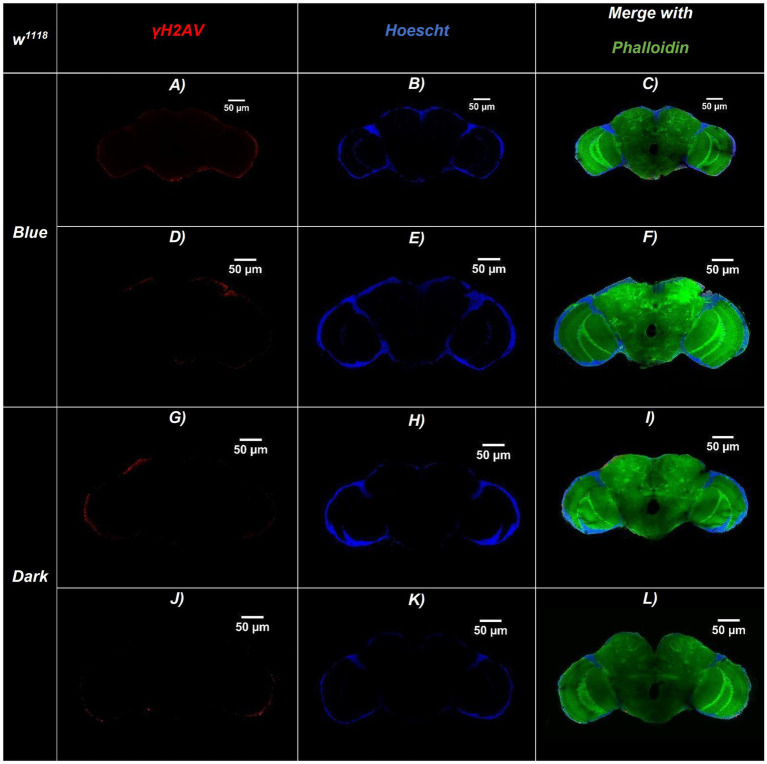
Representative confocal sections of adult *Drosophila* w^1118^ (wild-type) brains under blue-light or dark conditions. Brains were immunostained for DNA damage (γ-H2Av, red), nuclei (Hoechst, blue), and actin filaments (Phalloidin, green). **(A–F)** Brains exposed to continuous blue light (24 h/day for 20 days)—**(A,D)** γ-H2Av channel, **(B,E)** Hoechst channel, **(C,F)** Merged. **(G–L)** Brains maintained in constant darkness (24 h D/D): **(G,J)** γ-H2Av channel, **(H,K)** Hoechst channel, **(I,L)** Merged. Merged panels **(C,F,I,L)** show colocalization of all three markers, allowing visualization of brain structure and DNA damage. Brains exposed to blue light display a higher number of γ-H2Av–positive nuclei (red), indicating increased DNA damage compared to dark controls. Scale bars: 50 μm (all panels).

**Figure 5:**
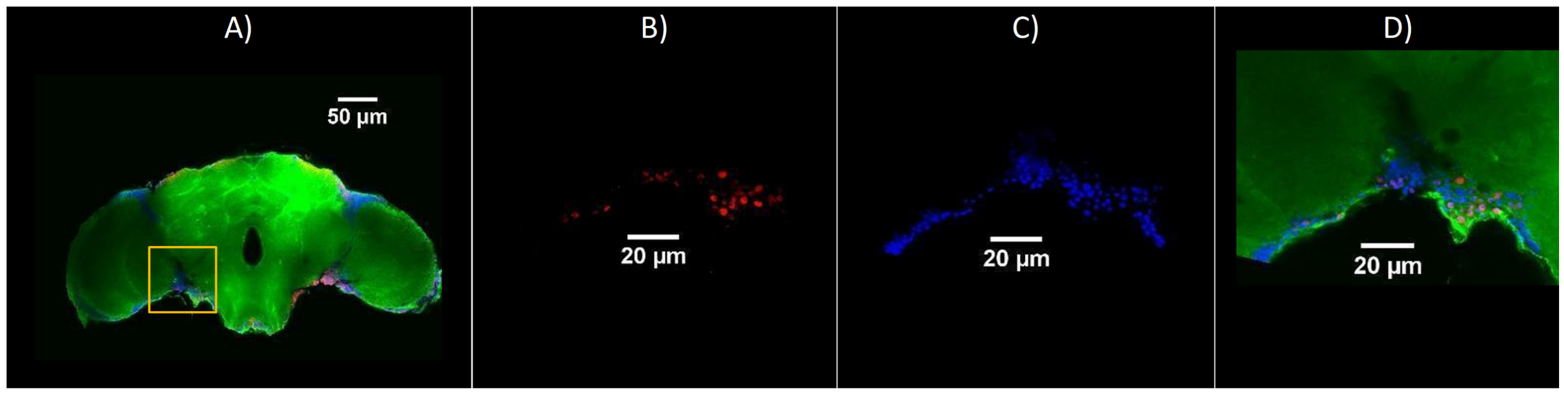
Representative confocal images showing the region of interest used for quantification of cellular staining in adult *Drosophila* brains. **(A)** Overview of a brain section with merged channels: γ-H2Av (red), Hoechst (blue), and Phalloidin (green); the yellow box indicates the region selected for analysis. Amplified views of the selected region—**(B)** γ-H2Av-positive cells (red, DNA damage), **(C)** Hoechst-stained nuclei (blue), **(D)** Merged image including Phalloidin (green), showing colocalization of nuclear and cytoskeletal markers with γ-H2Av signal. Scale bars: 50 μm (A), 20 μm **(B–D)**.

Representative confocal sections of adult *Drosophila* brains for the three genotypes are shown in Figures 2–4. Nuclei were labeled with Hoechst (blue), γ-H2Av–positive cells (indicating DNA damage) are shown in red, and actin fibers are stained with Phalloidin (green). Figure 5 illustrates an amplified region highlighting the merged staining.

**Figure 6:**
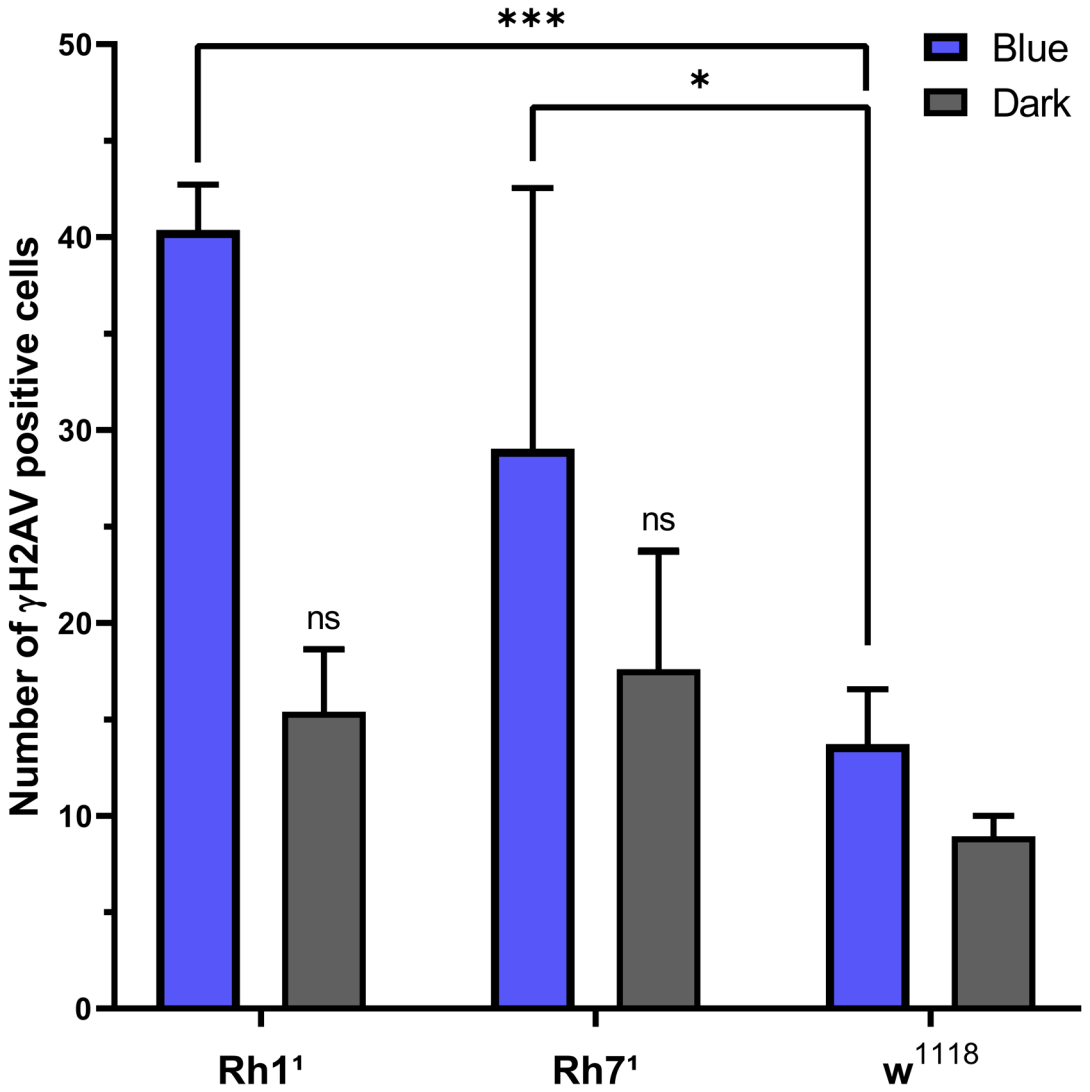
Proportion of γH2Av-positive cells in *Drosophila* brains under blue-light or dark conditions across genotypes (w^1118^, Rh1^1^, Rh7^1^). Values are expressed as mean ± SD. Statistical significance was determined by two-way ANOVA followed by Tukey’s multiple comparisons test (*n* = 3 brains per group). Under blue light, Rh1^1^ showed a significant increase in γ-H2Av–positive cells compared with w^1118^ (***p < 0.001), and Rh7^1^ also differed from w^1118^ (*p < 0.05). Only Rh1^1^ showed a significant difference between blue-light and dark conditions (***p < 0.001). ns = non-significant.

The bar chart in Figure 6 shows the number of γ-H2Av-positive cells in blue light-treated groups compared with dark-treated controls across the different genotypes. DNA damage was consistently higher under blue light in all strains, with Rh1^1^ flies showing the greatest increase compared to both w^1118^ and Rh7^1^. In contrast, under dark conditions, Rh1^1^ and Rh7^1^ flies exhibited similar numbers of γ-H2Av–positive cells, while w^1118^ flies showed a slight reduction (Figures 2–6).

### 3.2 Brain vacuolation is associated to Rh1 visual opsin deficiency

**Figure 7:**
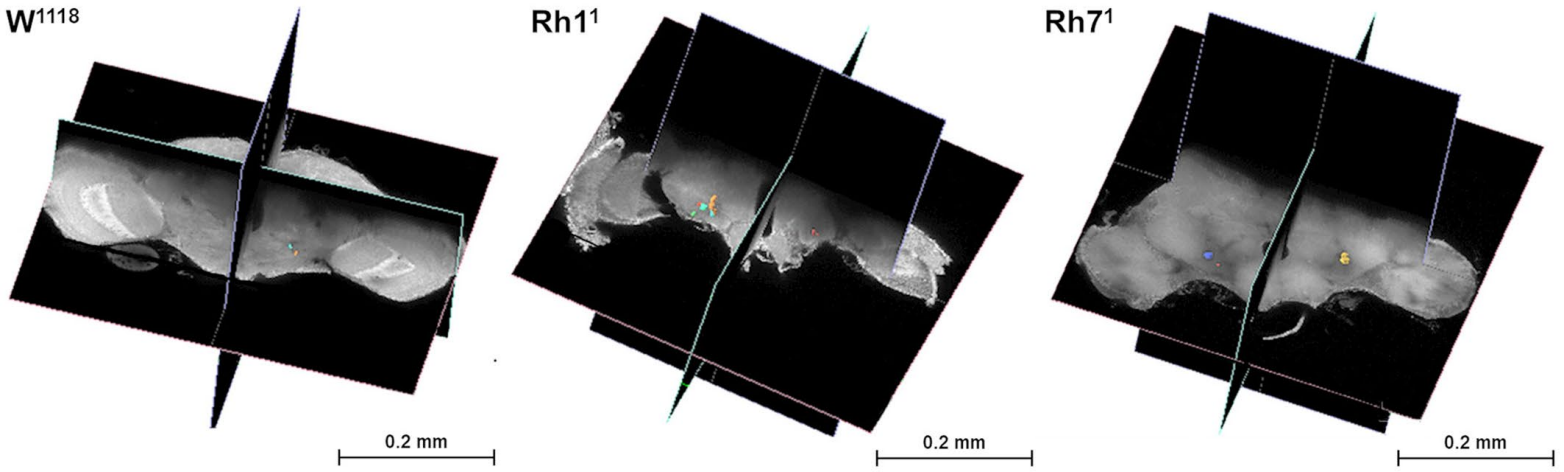
Grayscale, volumetric (3D) reconstructions of *Drosophila* brains from different genotypes (w^1118^, Rh1^1^, Rh7^1^) that were exposed to continuous blue light for 24 h a day for 20 days. Orthogonal cross-sections in the XY, YZ, and ZX planes (shown in green and blue) were generated using WebKnossos to enable volumetric inspection. Colored overlays highlight vacuole regions within the central brain, representing areas of tissue loss associated with neurodegeneration. Vacuoles were absent or sparse in w^1118^ brains, but were more frequently detected in Rh1^1^ brains and, to a lesser extent, in Rh7^1^ brains.

**Figure 8:**
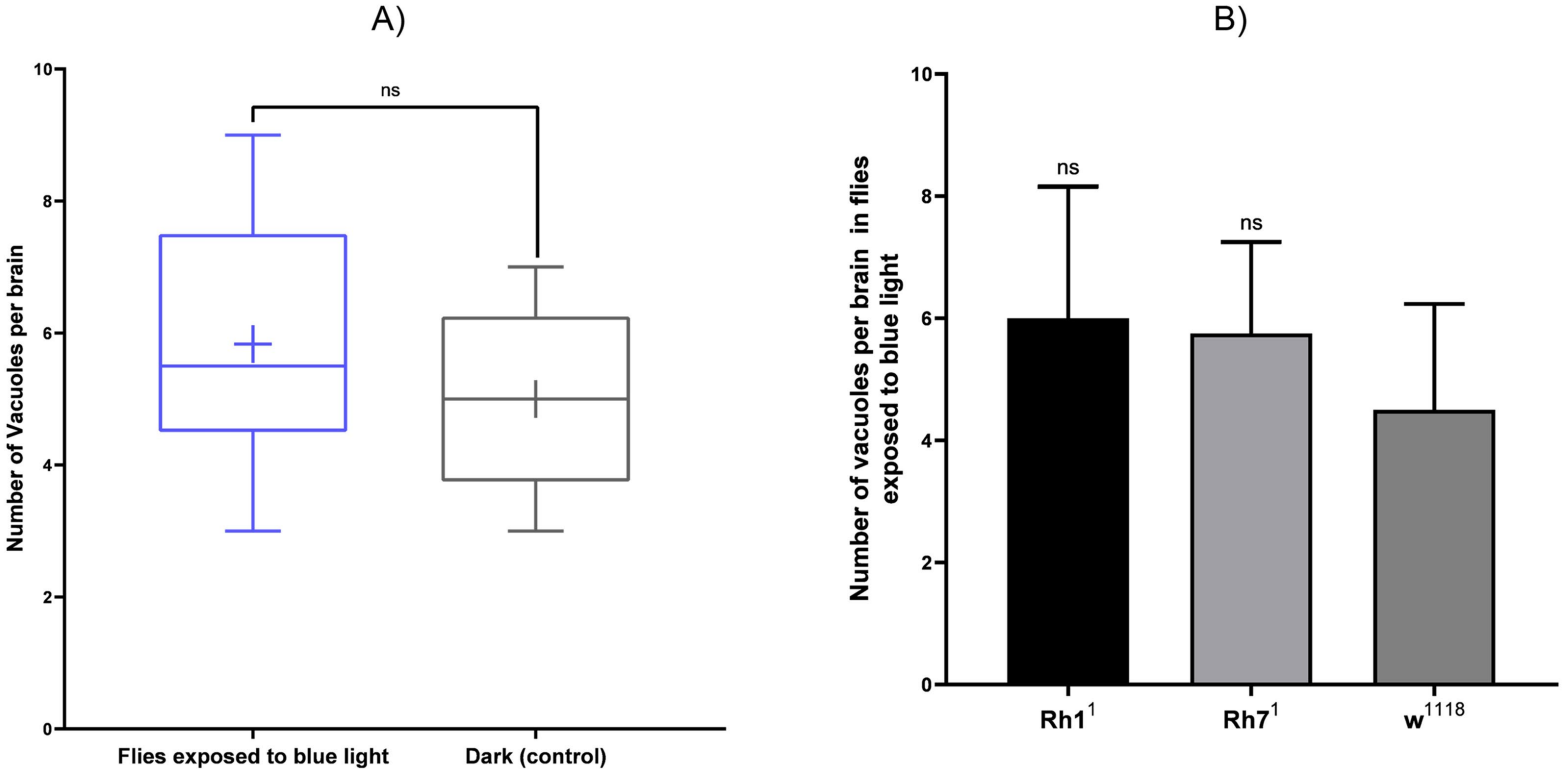
Quantification of brain vacuoles in *Drosophila* after continuous blue-light exposure (24 h/day, 20 days) compared with constant dark controls (24 h D/D). **(A)** Number of vacuoles per brain in flies exposed to blue light versus dark controls. **(B)** Comparison across genotypes (Rh1^1^, Rh7^1^, and w^1118^) under blue-light exposure. Values are expressed as mean ± SD (*n* = 3 brains per group). Statistical analysis was performed using the Mann–Whitney test for blue light versus dark (ns, *p* = 0.59) and Kruskal–Wallis followed by Dunn’s multiple comparisons test across genotypes (ns, *p* = 0.48). No significant differences were detected (ns).

Across the different genotypes, flies lacking the visual rhodopsin (Rh1^1^) exhibited a higher number of vacuoles in the central brain compared to the wild-type w^1118^ and the variant deprived of non-visual rhodopsin (Rh7^1^). Figure 7 shows grayscale, 3D volumetric images viewed in orthogonal cross-sections using the WebKnossos platform, which allows the identification of vacuoles in the central brain. The green bounding box and the black planes represent the slice views in three perpendicular orientations (XY, YZ, and ZX), allowing multiplane volume inspection. Quantification of all samples revealed a greater presence of vacuoles in flies exposed to blue light (Figures 7, 8).

## 4 Discussion

The relevance of studying neurodegeneration in *Drosophila melanogaster* has increased in recent years, as several studies have shown that excessive light exposure, especially at blue wavelength range (~450–490 nm), can induce neuron death. In this work, we investigated the effects of blue light exposure in *Drosophila melanogaster* flies with loss of function of visual and non-visual opsins. Our analyses show that ablation of different opsins (Rh1, a light-sensitive pigment, and Rh7, typically associated with non-visual functions) leads to distinct profiles of DNA damage (γ-H2Av) and vacuole formation in the brain of *Drosophila*. These findings support the hypotheses that each opsin contributes to photosensitivity and intracellular signaling in unique ways, culminating in distinct patterns of neurodegeneration under light exposure (25, 38). The Rh1^1^ strains showed increased DNA damage under blue light, compared to w^1118^ and Rh7^1^, supporting the notion that blocking the classical phototransduction pathway enhances DNA damage. The same pattern is observed across genotypes with respect to vacuole-associated neurodegeneration. The formation of neurodegeneration vacuoles in *Drosophila* indicates neuronal tissue loss and permanent brain damage (50), which can be assessed through the quantification of actin and synaptic protein filaments.

Quantitative analysis of Figures 6, 8 revealed an increase in γ-H2Av-positive cells and a modest rise in brain vacuolization in Rh1^1^ flies compared to w^1118^ controls. Compared to Rh7^1^ flies, the Rh1^1^ strain also showed a trend toward higher numbers of γ-H2Av-positive cells and slightly more vacuolization. These results suggest that the absence of the visual opsin Rh1^1^ may increase susceptibility to blue light-induced neurodegeneration, supporting a potential role of Rh1^1^ in mediating light-induced neurodegeneration in the central nervous system. Although the Rh7-ablated flies showed less damage under blue light than the Rh1-ablated ones, it was still greater than the w^1118^ control.

**Figure 9:**
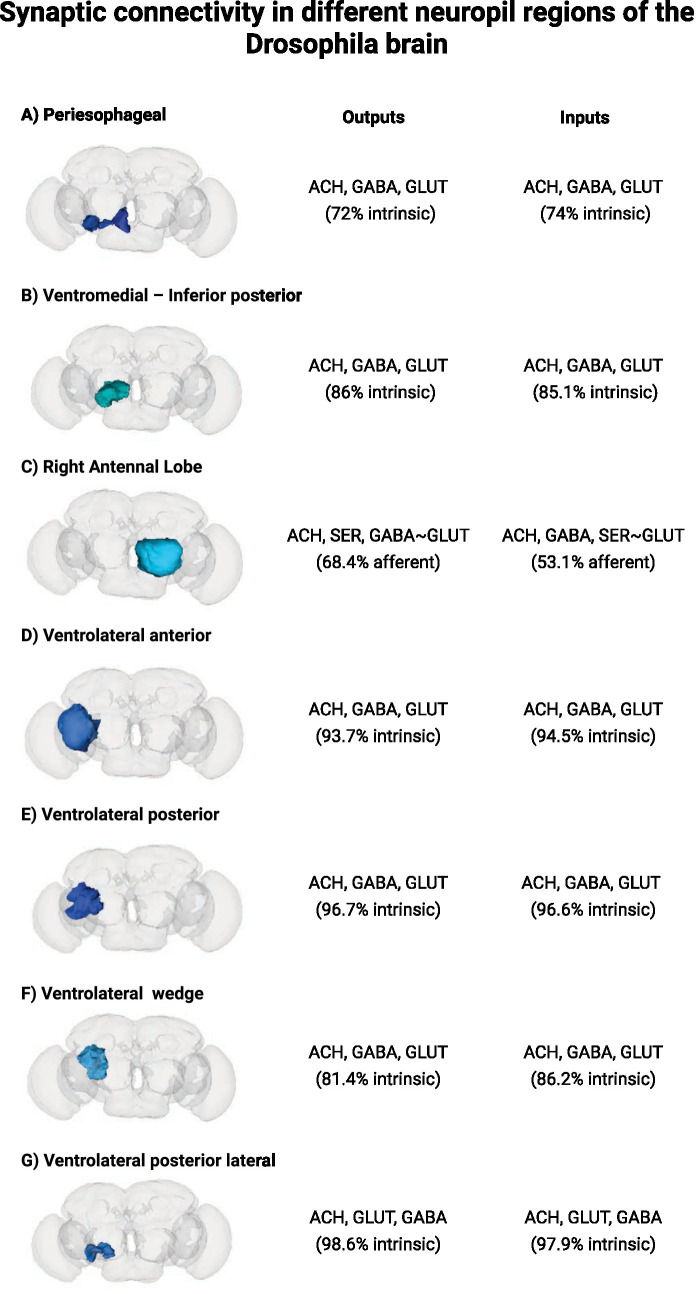
Characterization of synaptic connectivity in different neuropil regions of the *Drosophila* brain. Panels **(A-G)** highlight distinct brain regions: **(A)** Periesophageal neuropil, **(B)** Inferior posterior slope (IPS_L), **(C)** Right antennal lobe, **(D)** Anterior ventrolateral protocerebrum (AVLP_L), **(E)** Posterior ventrolateral protocerebrum (PVLP_L), **(F)** Wedge (WED_L), and **(G)** Posterior lateral protocerebrum (PLP_L). For each region, predominant neurotransmitter outputs and inputs are indicated, together with the proportion of intrinsic versus afferent connections (based on Codex database, Dorkenwald et al. (60)). Regions with lower intrinsic connectivity (e.g., periesophageal neuropil) are more reliant on afferent inputs and may be more vulnerable to vacuole formation, while highly intrinsic regions (e.g., PVLP_L, PLP_L) show greater local processing capacity and potentially lower vulnerability. Images adapted from codex.flywire.ai (60, 61).

Based on Figure 7, we identified and listed the *Drosophila* brain regions in which the vacuoles were observed. The analysis of the brain of *Drosophila* on the Codex platform (54) allowed us to highlight potential areas that are more vulnerable to neurodegeneration (Figure 9). The input regions represent the connections that transport information from other areas or sensory neurons to the brain. Output regions extend axonal connections from the neuropil to motor neurons or other brain areas.

Interestingly, vacuole was not equally distributed throughout the brain, but concentrated in specific regions, such as some ventrolateral regions of the protocerebrum (AVLP—Figure 9D, PVLP—Figure 9E) and periesophageal neuropils (WED—Figure 9F, PLP—Figure 9G), according to our volumetric analysis. Previous studies have linked the occurrence of vacuolization in specific regions of the *Drosophila* brain to local dysfunction of neurons and glial cells, which can culminate in severe functional impairment (55). When we cross-referenced our vacuole localization findings with the data available on the Codex platform for these regions (such as AVLP, PVLP, WED, PLP), we found an enrichment of excitatory (acetylcholine, glutamate) and inhibitory (GABA) neurotransmitter inputs and outputs, suggesting that these areas may be particularly vulnerable to oxidative stress when light-dependent processes—whether visual or non-visual—are compromised. This is consistent with studies showing that an imbalance between excitatory and inhibitory signaling can exacerbate degenerative processes. There is also evidence that excessive glutamatergic signaling contributes to neurodegenerative disorders in mammals, including Alzheimer’s disease (56). Furthermore, dysregulation of the excitatory/inhibitory balance, in part mediated by GABA, may accelerate neuronal degeneration and potentially contribute to cognitive and motor deficits.

These two groups were then sorted into intrinsic or afferent. Intrinsic connections are formed by local neurons that synapse within the same area without projecting signals to distant regions. A high percentage of intrinsic connectivity indicates strong self-regulation, with internal circuits responsible for processing information locally. In contrast, afferent connections bring sensory or modulatory information from other parts of the nervous system. Therefore, regions with high afferent connections may be more vulnerable to damage caused by light exposure, as connection loss can lead to more severe dysfunctions. The periesophageal neuropil (Figure 9A) has lower intrinsic connectivity among all the evaluated regions (approximately 72–74%), suggesting greater activation by external afferents associated with Rh7^1^ photo sensing.

The formation of vacuoles in the brains of flies possibly affects neurotransmitter balance and, consequently, behavior. The main neurotransmitters listed in the inputs and outputs of Figure 9 are Acetylcholine (ACH), GABA and Glutamate (GLUT), associated with anxious behavior (57), neurodegenerative processes (56), and ageing (58).

The right antennal lobe (Figure 9C) has the highest proportion of afferent connections (~68.4% output and 53.1% input), which may have been compromised by vacuole formation in all groups (Figure 7). Furthermore, regions such as the posterior ventrolateral (PVLP_L—Figure 9E) and posterior lateral protocerebrum (PLP_L—Figure 9E) have high intrinsic connectivity (>96%), indicating more preserved circuits and less vulnerability to external damage. The high number of vacuoles in those regions suggest that areas with greater dependence on external stimuli may be more vulnerable to light-induced neurodegeneration.

The occurrence of vacuolization in areas of high afferent connectivity indicates that the degeneration is affecting circuits that receive external inputs. This affects the sensory response and behavioral modulation of γ-H2Av-positive Rh1^1^ flies more prominently.

One hypothesis for the observed differences between the roles of visual and non-visual opsins is that, in situations of excessive blue light, neural pathways mediated by non-visual opsins (such as Rh7) are disturbed. This interaction could disrupt tissue homeostasis, leading to cumulative DNA damage and subsequent vacuolization. The absence of Rh1 would exacerbate this effect, perhaps due to the loss of protective feedback normally mediated by the retina, such as AMP-activated protein kinase (AMPK) (59), which results in increased vacuolization in the affected regions.

## 5 Limitations and future directions

This study offers valuable insights, though some limitations must be acknowledged. The limited number of biological replicates reduces statistical power; however, the consistency of results across replicates and methods supports the reliability of our findings.

Also, we employed a continuous exposure paradigm (24 h/day for 20 days), which, while useful to maximize cumulative dose and reveal clear effects, does not capture the potential influence of light–dark cycles, intermittent exposures, or different dose–response regimes. Furthermore, our analyses were focused on γ-H2Av as a marker of DNA damage and vacuolization as a marker of neurodegeneration; other relevant cellular pathways, including oxidative stress, apoptosis, synaptic integrity, and glial responses, were not assessed here. It would also be valuable to assess behavioral outcomes such as locomotor performance, and to explore rescue experiments in which Rh1 or Rh7 expression is restored. These approaches will help refine our understanding of how visual and non-visual opsins differentially modulate the neuronal response to blue light.

Future research should also include more detailed temporal analyses of damage progression throughout development and adulthood of animal models. Additionally, it will be essential to investigate the contribution of specific neural circuits and glial populations in these brain regions to elucidate how the absence of an opsin may activate distinct pathways of neurodegeneration in response to light.

## 6 Conclusion

Excessive blue light exposure is increasingly recognized as a public health concern, particularly due to widespread use of digital devices. Although the compound eye of *Drosophila* differs structurally from the camera-type eye of mammals, both systems rely on opsins as light-sensitive G-protein coupled receptors and share conserved downstream signaling cascades.

This study helps to elucidate the mechanisms by which different opsins modulate the cellular response to blue light and supports the idea that classical photoreceptors Rh1 influence processes beyond the retina. Additionally, our data suggests that non-visual opsins such as Rh7 may also participate in mediating photosensitivity within the brain. We show that visual opsins, specifically Rh1, are key mediators of blue light-induced DNA damage in flies’ brains. This aligns with mammalian studies reporting that excessive blue light can impair mitochondrial function, generate reactive oxygen species, and trigger DNA damage in retinal photoreceptors and ganglion cells, ultimately contributing to neurodegeneration and vision-related disorders such as glaucoma and age-related macular degeneration. Therefore, despite anatomical differences, the molecular vulnerability to high-energy blue light is conserved across species, underscoring the translational relevance of our findings and their potential impact on human health.

## Data Availability

The original contributions presented in the study are included in the article/supplementary material, further inquiries can be directed to the corresponding authors.
